# Patients’ Convergence of Mass and Interpersonal Communication on an Online Forum: Hybrid Methods Analysis

**DOI:** 10.2196/18303

**Published:** 2020-10-19

**Authors:** Remco Sanders, Theo B Araujo, Rens Vliegenthart, Mies C van Eenbergen, Julia C M van Weert, Annemiek J Linn

**Affiliations:** 1 Department of Communication Science, Amsterdam School of Communication Research Univeristy of Amsterdam Amsterdam Netherlands; 2 Department of Research Netherlands Comprehensive Cancer Organisation Utrecht Netherlands

**Keywords:** convergence, online health seeking, supervised machine learning, patient needs, machine learning, online forums, patients, media

## Abstract

**Background:**

Patients are increasingly taking an active role in their health. In doing so, they combine both mass and interpersonal media to gratify their cognitive and affective needs (ie, convergence). Owing to methodological challenges when studying convergence, a detailed view of how patients are using different types of media for needs fulfillment is lacking.

**Objective:**

The aim of this study was to obtain insight into the frequency of reported convergence, how convergence affects what posters write online, motives for posting, and the needs posters are trying to fulfill.

**Methods:**

Using a hybrid method of content analysis and supervised machine learning, this study used naturally available data to fill this research gap. We analyzed opening posts (N=1708) of an online forum targeting cancer patients and their relatives (Kanker.nl).

**Results:**

Nearly one-third of the forum opening posts contained signs of convergence in mass or interpersonal media. Posts containing mass media references disclosed less personal information and were more geared toward community enhancement and sharing experiences compared to posts without convergence. Furthermore, compared to posts without signs of convergence, posts that included interpersonal media references disclosed more personal information, and posters were more likely to ask for the experiences of fellow users to fulfill their needs. Within posts containing signs of convergence, posts including interpersonal media references reported fewer shortages of information, disclosed more information about the disease, and were more active in seeking other posters’ experiences compared to posts containing mass media references.

**Conclusions:**

The current study highlights the intertwining of media platforms for patients. The insights of this study can be used to adapt the health care system toward a new type of health information–seeking behavior in which one medium is not trusted to fulfill all needs. Instead, providers should incorporate the intertwinement of sources by providing patients with reliable websites and forums through which they can fulfill their needs.

## Introduction

### Background

Patients have the need to know and understand (ie, cognitive needs) and the need to feel acknowledged and understood (ie, affective needs) [1]. Currently, patients take an active role in the management of their health, and in doing so they combine mass and interpersonal communication to gratify their cognitive and affective needs (hereafter referred to collectively as “needs”) [2]. By using mass and interpersonal communication, patients engage in a process that is called “convergence” [3-5]. According to Kreps [3], convergence can be defined as “the sequence of impersonal to interpersonal interactions” (p. 519; type 1 convergence) or the “conduct of interpersonal and peer discussions about health-related issues in virtual discussion spaces of various kinds” (p. 521; type 2 convergence). We adapted and broadened this definition to include convergence between and within mass or interpersonal communication for the current study. That is, we consider convergence as a process in which either one mass communication source and one interpersonal communication source (intermedium convergence) or two mass communication sources (intramedium convergence) are being used to fulfill the user’s needs. For example, patients learn about their disease through a consultation with a medical expert (interpersonal communication) and then validate the advice of the medical expert by visiting a website (mass communication; intermedium convergence) [6-8]. An example of intramedium convergence is the use of treatment experience from a fellow patient’s blog post in one’s own blog post. 

These examples show how patients use mass and interpersonal communication to fulfill their needs. However, research into this topic often focuses on one singled-out communication source. As a consequence, current research does not provide insights into how communication sources affect each other and how needs differ depending on the sources used. Answering these questions is important since patients have different communication sources at their disposal. In particular, when it comes to online health information, patients often struggle to understand the complex information online, have difficulties in assessing whether the information is reliable, and might feel overwhelmed or experience information overload [9,10]. Therefore, patients and medical experts should work together in providing, validating, and discussing information. To determine which (online) source fits best, insights are needed on how patients combine sources and how the combination of sources affects needs. Ultimately, part of the costly and limited time of the medical expert could be used for referring to sources that can reliably fulfill part of the patients’ needs.

One explanation for the research gap on how patients combine multiple sources to fulfill their needs can be found in the methodological challenges faced when studying this process. The scarce research in health communication in which both mass and interpersonal communication are taken into account tends to rely on more traditional research methods such as surveys [11,12], interviews [13], and focus groups [14].These methods can be affected by selection bias, recall bias, and social desirability [11]. By using a hybrid method consisting of content analysis and supervised machine learning (SML), the limitations of these traditional methods can be surmounted [15]. The hybrid method combines a content analysis of real-life communication and SML. We use natural unsolicited data (ie, data that are not obtained as part of research but are instead compiled by the user writing at the time their needs arose). The benefit of this method is that large amounts of already existing natural data can be used to study the current topic.

A good starting point is the analysis of data from online health forums targeting patients. Forums provide a natural database of people’s online activities. In forum opening posts, posters often provide information about their situation at the time of writing, which often includes previously used communication (ie, signs of convergence), the outcome of this communication effort (ie, motives to start the forum post), and the needs they are trying to fulfill. Additionally, background information about the poster is often included (eg, stage and type of the disease, and whether the poster him/herself or a family member has been diagnosed with the disease) [3,16]. Thus, by using this hybrid method, we are able to gain more insight into how often patients combine mass and interpersonal communication, the reason as to why they engage in convergence, which need they are trying to fulfill, and whether the content of forum posts differs based on the communication sources they used prior to writing the post. The following research aim was central in the present study: What signs of convergence can be detected in forum opening posts, how frequently does convergence occur, what kinds of needs are patients trying to fulfill by engaging in convergence, and how do forum post characteristics (ie, motives, information about the poster, and needs) differ for different kinds of convergence?

In this study, we used forums in the context of cancer. Cancer patients are confronted with many questions and uncertainties during their illness [17,18]. Furthermore, online platforms such as forums and interpersonal communication with a health care provider are the two most important sources of information for cancer patients [19].

### Interpersonal Communication

In general, most patients consider medical experts to be the most trusted source of information [20]. In a review, Shea-Budgell and colleagues [20] highlighted that patients place a high level of trust in medical experts owing to their expertise on topics that patients find most important, including treatment, screening, testing, and detection. Furthermore, their medical and informational training and, to a lesser degree, the emotional support they provide during a consultation are mentioned as factors that instill confidence [21-23]. Nevertheless, between 40% and 90% of patients report unmet needs after their consultation with a medical expert [24,25]. Multiple reasons can be given for these unmet needs. Patient-related reasons include unmentioned concerns, a lack of trust in a particular medical expert, and information overload [26]. Examples of medical expert–related concerns are time constraints and a lack of experience [27]. Therefore, patients also rely on other sources for needs fulfillment, such as online forums. Patients expect their medical experts to discuss the content they found via other sources and to offer their professional take on it [28]. By discussing online health information with their provider, patients engage in intramedium convergence.

### Online Forums

Online forums are often used by patients and their relatives and can be considered as virtual communities. Virtual communities exist in many different areas, cover many topics, and connect groups with a variety of shared characteristics. In this study, we adopt the definition of Rheingold [29], who states that virtual communities are “social aggregations that emerge from the Net when enough people carry on those public discussions long enough, with sufficient human feeling, to form webs of personal relationships in cyberspace.” Many patients encounter various needs and use multiple communication sources to fulfill these needs. As a result, these patients are active in online forums while also often having contact with other sources such as medical experts [30]. Patients use these platforms to gain understanding about their disease but also to connect and exchange experiences and support with others in comparable situations [7,8,31]. By encouraging and enabling active participation (eg, by opening a thread on a topic that is personally relevant), forums have the potential to provide different types of support to the user, such as to receive the support of their peers, to feel empowered through information provision, and to recognize themselves in stories from peers and thereby feel less isolated [11,32]. The malleability of forums in addressing patients’ needs and the ability to do this at any time might be a key reason why patients turn to these forums.

### Convergence and Underlying Motives

Many patients decide to combine multiple sources to fulfill their needs. Generally, 25% to 83% of patients search for online health information before or after a consultation with their medical expert [33-36]. Patients seem to use online health information in addition to a consultation to prepare themselves [13], to complement the information given by the medical expert [13,24,25], as well as to validate or challenge the information given by the medical expert [13].

To understand why patients use multiple sources, the optimal matching model can be used [37,38]. This model states that to fulfill patients’ needs, these needs should be matched with the right type of support. For example, if the patient feels the need to prepare for a consultation or wants to complement, validate, or challenge the information that is given by the medical expert, this need can be fulfilled by gathering factual information from other sources. By contrast, if the patient feels lost and alone, this need might not be fulfilled by receiving information about the upcoming treatment but rather by receiving emotional support that helps with the emotional aspects of being sick. According to the optimal matching model, patients actively choose the communication channel that they believe has the highest potential to fulfill their current needs. A patient who feels that they should prepare for the consultation is more likely to choose online medical libraries to fulfill these needs, whereas a patient who feels lost will more likely turn to online health forums and blogs, on which interaction with fellow patients is possible [3,31,39-41]. This exploratory research contributes to the optimal matching theory by identifying whether and how patients fulfill their needs by using multiple sources at their disposal and how these sources are intertwined.

### Research Questions

In summary, we believe that forum posts provide a natural database of peoples’ communication activities, and these forum posts can offer an opportunity to gain a better understanding of the interplay between communication channels. Therefore, our first research question (RQ1) is proposed as follows: What is the frequency of signs of convergence in forum opening posts? Furthermore, these forum posts can provide a natural registration of the motives for using different media (eg, after seeing a doctor or reading online health information) to fulfill specific needs. Therefore, research question 2 (RQ2) is proposed as follows: What needs are patients trying to fulfill by opening a forum post? By using a hybrid method to analyze these forums, we are able to also capture other relevant information such as the disclosure of information about the poster and the motive for posting [3,16]. These aspects are important for providing insight into how users in different situations gratify their needs or the needs of relatives by using multiple sources. Therefore, we propose research question 3 (RQ3) as follows: How do motives (3a), information about the poster (3b), and the needs (3c) differ for different types of convergence?

## Methods

### Study Design

We used a hybrid method consisting of a classic social science method (ie, the framework method [42]) and a newer computational social science method (ie, SML). The benefit of this approach is two-fold. First, this method allows us to combine unique features from both approaches. On the one hand, the framework method starts from a theory-based codebook (ie, the use of sensitizing concepts) and is then further developed through an iterative process of (open) coding on a subsample of the data. On the other hand, the coded subsample can then be used to label the whole sample with codes and categories using SML, thereby allowing us to move from open-coded data on a subsample to data that are suitable for quantitative analysis based on the complete dataset, allowing researchers to analyze sample sizes that were impossible to code manually. Second, SML allows us, as well as other researchers and practitioners, to (re)use the trained model on a different dataset or for practical applications. The reuse of the algorithms makes cost-efficient longitudinal research into convergence possible since the models can automatically and consciously be applied to new data.

### Data

We used data retrieved from cancer patients and relatives on the online forum Kanker.nl [43] in the Netherlands. Cancer is the most common disease, with a yearly incidence rate of 439.2 per 100,000 men and women (18,078,567 in 2018) and a yearly rate of 163.5 per 100,000 people dying from cancer (9,555,027 in 2018) [44]. Furthermore, cancer patients and their relatives experience multiple visits with a medical expert and face many questions and uncertainties. In the Netherlands, Kanker.nl is one of the largest and best-known Dutch websites for cancer-related information within an online community [43].

Users are required to register and must provide their name and a valid email address. Participants of all platforms within Kanker.nl give (standard) consent for using their data for research when they register. Ethical approval for the current study was provided by the ethical committee of University of Amsterdam (2016-PC-7547).

For the complete dataset, first, all forum entries (N=9573) were extracted. Second, only the opening posts of the threads were selected (n=1708). The opening posts were chosen because they are most likely to contain a description of the situation and the need the user wants to fulfill. The median number of words for each thread opening was 608.05 (range 3-20,649). The threads were created between April 2013 and November 2016.

### Phase 1: Framework Method

Of all 1708 thread openings, a random sample of 306 posts (17.92%) was manually coded by the first author (RS) in multiple iterations. First, using two sensitizing concepts derived from the literature (ie, information sources and motives for searching health information), 100 posts were open-coded on signs of convergence (RQ1), motives for opening a forum thread (RQ2), sought-after need (RQ3), and personal characteristics (RQ3); in total, 583 different codes were used for the constructs needed to answer RQ2 and RQ3. Second, these open codes were merged into overarching, latent categories. For example, the distinctions between different medical experts such as general practitioners, oncologists, and nurses were merged into a medical expert category. Third, the codebook and categories were evaluated on completeness during research meetings with the coauthors (AL, RV, JvW). As a result, several categories were merged again, and more specific categories were added. This process resulted in the following categories: convergence, motive for posting, information on poster, and needs (see Table 1 for the codebook). Fourth, the updated codebook was evaluated by the first author and a trained coder (RS and MB) who double-coded 20% of the sample. The intercoder reliability was good (Lotus range 0.98-1.00); Lotus and standardized Lotus scores are displayed per category in Table 2. Fifth, all 306 exported thread openings were manually coded by the first author (MB) using the codebook. Finally, the manually coded posts were transformed by the first author (MB) into binary variables to be used in the second phase.

**Table 1:** Overview of categories and codes analyzed.

Codes/classifiers		Description	Example	
**Convergence**				
	Mass communication	(1) online and (2) offline media. Mass communication was coded as such when communication channels such as internet sources (ie, online) and television and newspapers (ie, offline) were mentioned.		“[…] on a website I read that […]”; “This article in today’s newspaper […]”
	Interpersonal communication	Interpersonal communication was coded as such when one of the following communication sources was mentioned: (1) medical experts (eg, general practitioner, nurse, surgeon), (2) fellow patients, (3) family members, or (4) others.		“My doctor told me that […]”; “According to my mother […]”
	No media	Posts contained no references to other media.		“I was wondering if any of you knows something about […]”
**Motive for posting**		For posts containing mass media or interpersonal media references.		
	Conflict	Information is received from a medium that is contradictory to information previously acquired from another medium or contradictory to held beliefs. Resolving this discrepancy is a motive to open a forum thread.		“[…] My doctor told me this treatment is not an option for me, but I heard lots of stories that it was successful […]”.
	Shortage of information	Poster indicates that (s)he received little or no information regarding a topic. To fill this information gap, a forum thread is opened.		“[…] There was no time during the consultation to discuss the trajectory of this alternative […]”.
	New question	Poster indicates that as a result of information provided during the mentioned communication effort, (s)he has new (follow-up) questions. Answering these questions is a motive for opening a thread.		“[...] the doctor mentioned this medicine can have a lot of side effects, but is it common to experience them?”
	Sharing information	Poster wants to share the information/content that was received during the previous communication effort on the forum.		“I read this [website] and thought it might be useful for all of you”.
**Information on poster**				
	Disease or treatment information	Specific stages of the disease (eg, stage one) or treatment (eg, after surgery) were described in the post.		“[...] I'm diagnosed with stage one breast cancer”; “[...] After surgery I noticed that [...]”
	Time indication disease or treatment	Diseases or treatments that were mentioned at the “disease or treatment information” stage are further specified with a time indicator.		“One year after my surgery, I went back to the hospital [...]”
	Type of cancer	Type of cancer is mentioned by the poster.		“I have been diagnosed with lung cancer”
	Cancer in the surrounding community	The poster him/herself did not receive a diagnosis but someone close to him or her has.		“My husband has been sick for a few years now, I wonder [...]”
**Needs**				
	Community building	The post is meant as a conversational starter, including rhetorical questions and a direct call for discussion. All without asking for experiences or advice.		“What is your opinion about the quality of care in the Netherlands?”
	Sharing experiences	The poster is sharing experiences about the treatment or psychosocial aspects surrounding (living with) cancer.		“For me, this kind of treatment worked very well without too many side effects” or “For me, it worked to limit the number of social activities in a week”.
	Asking experiences	The poster invites other forum members to share their experiences about a certain topic.		“Who has experience with this?”
	Asking for information	The poster asks for more information about a certain topic or asks for referrals to sources where this information can be obtained.		“Who knows where I can find more information about this?”

**Table 2:** Intercoder reliability using Lotus and standardized Lotus (S-Lotus) coefficients per variable.

Concept			Lotus	S-Lotus
**Convergence**				
		Mass communication	1.00	1.00
		Interpersonal communication	1.00	1.00
		No media	1.00	1.00
**Specification of convergence**				
		Online	1.00	1.00
		Offline	1.00	1.00
		Medical expert	1.00	1.00
		Fellow patients	1.00	1.00
		Family members	1.00	1.00
		Others	1.00	1.00
**Motive for posting**				
	Conflict		1.00	1.00
	Shortage of information		1.00	1.00
	New question		0.98	0.96
	Sharing information		0.98	0.96
**Information on poster**				
	Disease or treatment information		1.00	1.00
	Time indication disease or treatment		1.00	1.00
	Type of cancer		0.99	0.97
	Cancer in the surrounding community		1.00	1.00
**Needs**				
	Community building		1.00	1.00
	Sharing experience		0.99	0.97
	Asking experience		1.00	1.00
	Asking information		0.99	0.97

#### Codebook

**Figure 1:**
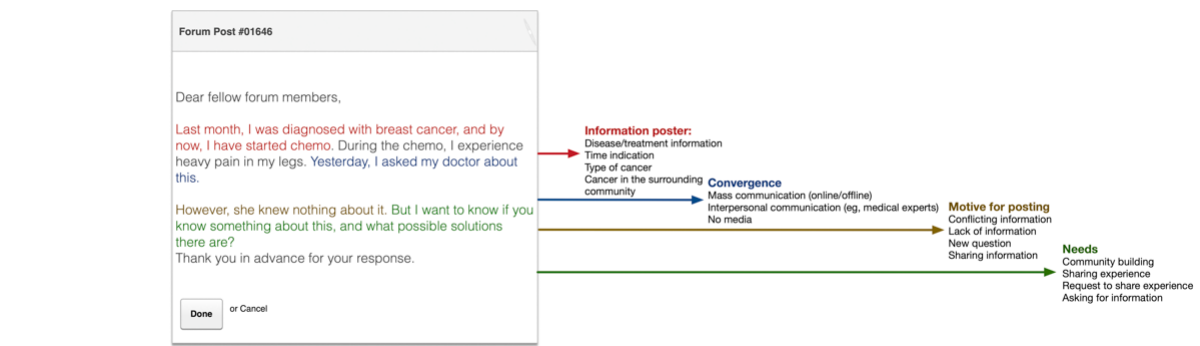
Example of extraction of concepts from forum posts.

Table 1 contains the categories and codes that were coded during the SML phase. These variables were coded as 0 (not present) or 1 (present). Figure 1 shows a fictitious example of the extracted concepts from the forum opening posts.

### Phase 2: SML

We used SML to train classifiers for the references to mass or interpersonal communication (see Multimedia Appendix 1 for a detailed description of the SML phase). A sample of 685 manually coded opening posts (in two rounds) was used as input for SML. This sample was split into a training set (n=548) and a test set (n=137) using an 80-20 split. Using Scikit-Learn [45], the data were preprocessed (see Multimedia Appendix 1 for a detailed overview), and the classifiers were trained using different algorithms such as support vector classification, stochastic gradient descent, multinomial naïve Bayes, gradient boosting, and passive aggressive classifier. This was done to evaluate which algorithm would have the best performance for each concept it was trained to predict. In this process, we adopted a grid search strategy, which tests different combinations of parameters for each algorithm as well as different options for preprocessing the data.

The quality of the classifiers was assessed based on precision, recall, and F1 scores for their predictions of cases in which the category was present (ie, for cases in which the reference to mass or interpersonal communication was 1). Precision gives the proportion of the automatically assigned labels that correspond with the human-labeled data. Recall gives the proportion of the true labels that are found automatically. This often results in a tradeoff between the scores of precision and recall; for example, in cases of higher recall, the chance that some of the recalled data are false positives grows, and the precision score consequently goes down. F1 scores are the harmonic mean of the recall and precision. Stochastic gradient descent proved to have the best performance in predicting the classifiers convergence mass media and convergence interpersonal media (recall_interpersonal_=0.76, precision_interpersonal_=0.96, F1=0.85; recall_massmedia_=0.86, precision_massmedia_=0.92, F1=0.89). See Multimedia Appendix 2 for the complete confusion matrix. These classifiers were applied to the complete dataset of opening posts (N=1708) to create a subsample of opening posts that were likely to contain signs of convergence (n=771, 45.14%).

### Phase 3: Manual Coding Convergence Posts

To ensure the validity of the automatically assigned classifiers, the created subsample of posts was checked for correctness. Of the automatically labeled posts containing signs of convergence (n=771), 245 posts (31.78%) did not contain signs of convergence after manually checking, and were coded as “no media.” Next, the subsample of posts was manually coded by the first author (MB) using the codebook for the remaining categories (see Table 1). 

### Analysis

Before running the analysis, all independent variables were tested on possible issues due to multicollinearity. Only issues concerning time since diagnosis, stage of the disease, and type of disease (r_time-stage_=0.82, r_time-type_=0.92) were found; thus, these items were taken together as “disclosure of information about the disease” (eigenvalue=2.71; *R*^2^=0.90; α=.95).

RQ1 and RQ2 were answered using descriptive analyses. To compare the outcomes on the dependent variables between posts containing different signs of convergence (RQ3), two multinomial logistic regression analyses were conducted. In these two analyses, the referenced communication channels were the dependent variables (ie, no media, mass communication, or interpersonal communication). The first regression used no media as the reference category with information on poster and needs as the independent variables. Since the category “motive” was not applicable to the “no mass” or “interpersonal media” category, motive was omitted from this analysis. However, to pinpoint the differences between posts containing signs of convergence, motive, information on poster, and needs were included in the second regression analysis, in which mass communication was used as the reference category. The outcomes of these analyses are displayed as the odds ratios (OR). An *R* value of 1 indicates no differences in probability between the groups compared, whereas a value >1 represents an increased probability and a value <1 represents a decreased probability [46].

## Results

### Signs of Convergence in Forum Posts

The results showed that 30.80% of the complete sample of forum opening posts (n=526) contained signs of convergence. These were divided into mass communication (324/526, 61.6%) and interpersonal communication (202/526, 38.4%). In the following sections, these categories will be described in more depth.

Of all mass communication references (n=324), 274 (84.6%) referred to online sources (eg, other members’ profiles or blogs, news media articles concerning cancer [patients], and health information websites). Of all posts with mass communication references, 214/324 (66.1%) posts contained references to a website. Offline mass communication was referenced 49 (15.1%) times, which included references to printed newspapers, books, and television.

Of all interpersonal communication posts (n=202), 162 references (80.2%) were made to medical experts (eg, oncologists, nurses, and general practitioners) and 14 references (6.9%) were made to family members. These include family members who either had personal experiences with the disease or provided information they received via other sources. Fellow patients who provided information offline were referenced 3 times (1.5%) and 22 references (10.9%) were made to communication events with other people. Often, these events consisted of work-related relationships (eg, employers, insurers, and rehabilitation agents).

With respect to RQ1, almost one-third of all forum opening posts contained signs of convergence and thus included references to either mass or interpersonal communication. When referencing mass communication, mainly online sources were mentioned, whereas for interpersonal communication, medical experts were most often mentioned.

### Posters’ Needs

Of all 771 opening posts containing signs of convergence, 344 posts (44.6%) represented the need of asking for experiences regarding a specific treatment (eg, medicine, procedure) or experiences regarding (dealing with) the (emotional) effects of living with cancer (ie, dealing with side effects, reintegration into society, and body image). This was followed by forum opening posts related to community building (266/771, 34.5%). In these cases, the poster started a discussion about a particular topic such as developments in the medical sector, with or without a URL to a news story (online). The third-largest need to open a forum thread was to share one’s personal experience. Overall, 143/771 (18.6%) of the posts featured this need. Finally, in 72 of the 771 opening posts (9.3%), the poster directly asked for sources to find more factual information on a particular topic such as (alternative) treatment options. Therefore, to answer RQ2, the main need for patients to be fulfilled, as reflected in forum openings post, is that of asking for information related to experiences. This need is followed by that of enhancing the community, sharing one’s experiences, and asking for factual information.

### Differences in Posts for Different Kinds of Convergence

The first multinomial logistic regression model contained the variables from the categories information on poster and needs (adjusted *R*^2^=0.30, χ^2^_12_= –563.27, *P*<.001; Table 3). In posts referencing mass communication, the disclosure of disease-related information was 89% less likely to occur compared to posts that did not include a reference to mass communication. In contrast, posts including references to interpersonal communication had a 156% higher likelihood of featuring the disclosure of disease-related information (Table 3) compared to posts containing no references to media. These outcomes mean that the chance of disclosing disease-related information in forum posts in which interpersonal communication is mentioned is higher compared to that of forum posts with no signs of convergence and is lower for posts that include references to mass communication.

When considering the needs posters might have for opening a forum thread, differences in needs within different types of convergence were found. Higher likelihoods were found for posts including references to mass communication compared to posts containing no signs of convergence for the needs: community building, sharing experiences, and asking for information. This means that after mass communication exposure, posts have a 373% higher likelihood of containing the need to share the post for community building, a 291% higher likelihood of containing the need to share one’s experience with others, and a 188% higher likelihood of asking for more information compared to no exposure to mass or interpersonal communication. Posts containing references to interpersonal communication had a 268% higher likelihood of displaying the need to ask fellow patients for their experiences compared to posts containing no signs of convergence.

**Table 3:** Differences between posts containing signs of convergence and posts without (reference category=no media).

Variable		Mass			Interpersonal		
		OR^a^	*P* value	95% CI	OR	*P* value	95% CI
**Information on poster**							
	Disclosure of information about the disease	0.11	<.001	0.06-0.20	2.56	.009	1.26-5.20
	Cancer in surrounding	1.08	.85	0.50-2.29	1.51	.10	0.92-2.49
**Needs**							
	Community building	4.73	.002	0.92-2.49	0.21	.06	0.04-1.10
	Sharing experiences	3.91	.003	1.59-9.59	1.71	.21	0.75-3.89
	Asking for experiences	1.30	.58	0.52-3.30	3.68	.004	1.50-8.99
	Asking for information	2.88	.04	1.04-7.98	1.91	.23	0.67-5.46

^a^OR: odds ratio.

The second multinomial logistic regression models contained the variables from the categories motive, information on poster, and needs (adjusted *R*^2^=0.77, χ^2^_20_= –185.40, *P*<.001; Table 4). Within the category motive, in posts referencing interpersonal communication, a shortage of information was 81% less likely to be the reported outcome of the communication effort compared to posts referencing mass communication. Furthermore, within the category information on poster, posts referencing interpersonal communication were 2015% more likely to disclose information about the disease compared to posts referencing mass communication. Within the category needs, posts containing interpersonal communication were 93% less likely to display community building as a need of the post compared to posts referencing mass communication. Furthermore, posts referencing interpersonal communication had a 227% higher likelihood of asking for other posters’ experiences compared to posts referencing mass communication (OR 3.27, *P*=.04).

To answer RQ3, compared to intramedium convergence, intermedium convergence posts are less likely to be motivated by a shortage of information and are more likely to contain information about the poster’s condition. Furthermore, again compared to intramedium convergence, intermedium convergence is more likely to display the need for experiences and is less likely to exhibit a need for community building.

**Table 4:** Differences between posts containing signs of interpersonal convergence (reference category=mass communication).

Variable		OR^a^	*P* value	95% CI
**Motive**				
	Conflict	2.77	.07	0.92-8.29
	Shortage of information	0.19	.006	0.06-0.63
				
	New questions	0.83	.69	0.30-2.23
	Sharing of information	0.39	.11	0.12-1.24
**Information on poster**				
	Disclosure of information about the disease	21.15	<.001	9.39-47.62
	Cancer in surrounding	1.49	.36	0.64-3.47
**Needs**				
	Community building	0.07	.004	0.01-0.44
	Sharing experiences	0.65	.47	0.20-2.10
	Asking for experience	3.27	.04	1.01-10.57
	Asking for information	0.77	.70	0.20-2.90

^a^OR: odds ratio.

## Discussion

### Principal Findings

This study provides more insight into (the occurrence of) convergence using natural unsolicited data. Overall, intramedium and intermedium convergence resulted in posts containing different content and aiming to fulfill different needs. We found that nearly one-third of all forum opening posts in our sample contained signs of convergence by referencing either mass or interpersonal communication in the post. For intramedium convergence, online sources such as websites, forums, and online news articles were most often mentioned, frequently accompanied by a link to that source. In this way, posters seem to fulfill their need to help build the online community and initiate a discussion or to share experiences. Posts containing intermedium convergence often included references to a consultation with a medical expert. In these posts, users reported less shortage of information, disclosed more about themselves, and asked for more experiences from other users compared to posts containing intramedium convergence.

Our findings further emphasize the frequency of reported convergence and how intertwined these sources are. The main interpersonal communication source that was mentioned in the posts was that of a medical expert. This outcome is in line with previous research in which the medical expert, together with the internet, is named as the most important source of information for patients [19,23,47]. We found that one-third of the posts contained signs of convergence. The number of patients who use more than one medium is likely to be higher for two reasons. First, we only looked at specific types of convergence occurring in forum posts; however, based on previous research (eg, [35]), we know that signs of convergence also occur at the medical encounter and that different types of convergence exist. For example, during medical encounters, patients could discuss a forum they have read before the consultation and thus engage in intermedium convergence (online forum-medical expert) or engage in intramedium convergence (ie, medical expert-medical expert) by referencing a medical expert during the consultation who provided a second opinion.

Second, we only coded explicit signs of convergences, whereas previous research also shows that patients implicitly mention different sources [48]. One aspect that is unique to this study is that although previous studies often examined both sources independently, the current results show how interdependent these sources are and how they are likely to continue to merge in the future. For example, a poster who recently had an appointment with a medical expert may have received a lot of information (convergence). After interpersonal communication, there is a lower likelihood that the patient experienced a shortage of information (motive). However, the patient might have missed information about how other patients experienced the situation, which motivates the patient to go online, write about their situation, and ask fellow patients for their experiences (need). According to the optimal matching theory [38], patients actively choose a medium that likely fulfills their needs.

In the context of support, some patients actively start participating in forums to find information that only fellow patients can provide—their experiences [14,49,50]. Our results also highlight the importance and added value of studying information sources in an interdependent context instead of independently. In light of the increased availability of different types of information on platforms, the internet seems to be a promising venue to fulfill needs that are not fulfilled during a consultation. Taking the notion of the optimal matching theory further, one could argue that it should not be a problem if patients report unmet needs based on their exposure to one medium, as another medium might be better able to fulfill these unmet needs. However, the medical expert and patient should work together to make sure credible sources of information are known and available to the patient to fulfill their needs.

Based on our results, posters seem to require information provided by other patients combined with the information provided by the medical experts. Forums can be used to gain access to the experiences of fellow patients without the medical expert being an intermediary in this process. Users thereby benefit from both the expertise garnered during consultations with the medical expert and the experiences of fellow patients [51]. Eysenbach and colleagues [50] already highlighted that providing, receiving, and reading experiences from fellow patients is one of the main functions of social support communities. The current study shows how patients use health forums in a broader context of multiple available sources.

Because websites are easily shared and embedded in online tools such as online forums, the current study found many references to mass communication in general and online sources in particular. Mass communication is likely to be shared with members of the community to sustain and to inform the community through what is called “community building.” Community building creates a feeling of being part of a community and therefore fights the feeling of being alone, which in turn can emotionally support the patient [52].

### Limitations and Future Research

We posited that using a hybrid method on natural data could be a useful tool in meeting the challenges faced in studying convergence (ie, circular process, biased data when trusted on solicited recall data). Although we successfully analyzed indicators for convergence using forum data, some shortcomings must be acknowledged to advance future research. Despite the merits in using unsolicited data, not all aspects of convergence could be studied. First, we could only detect explicit signs of convergence. It would be a safe assumption to imagine convergence occurring in implicit ways as well, such as by simply posting a question without stating the events leading up to the post. Furthermore, convergence could only be measured when mass or interpersonal communication led to posting on a forum. However, posting online or reading posts and responding to these posts could lead to convergence elsewhere. By only studying online forum posts on one particular website, these types of convergence could not be measured. Although this would result in an underestimation of convergence instead of an overestimation, future research could address these types of convergence. Content analysis (on videotaped consultations) can, for instance, be combined with surveys to investigate patients’ (unmet) needs when they communicate and to gain insight into how patients use communication sources to cope with their needs. The online environment would be a logical place to administer these surveys since this environment does not require actual tracking; instead, log data and prompted surveys could minimize intrusion and reliance on recall. Finally, using natural data restricted the possibility to control for differences in personal characteristics of the poster because these variables are not known. Based on previous studies, we know that the way patients use online forums changes over time [53]. We did not account for these individual differences. Future studies could gather data from multiple forum messages and profiles to extract information on the time of diagnosis, number of posts by the user, and type of disease to gain insight into these concepts.

SML was applied to create a subsample of posts containing signs of convergence. This approach resulted in a significantly smaller sample that had to be manually coded. If studies are interested in latent communication concepts such as the needs or motives of patients, researchers should take into account the time and effort needed to code a substantial part of their data as input for the SML, still without a guarantee that these latent construct can be reliably predicted. In an early phase of their study, researchers should decide on the role of SML in their project based on the number of positive cases per classifier and the initial SML results. Instead of coding a large portion of their data in the hope to obtain reliable classifiers for all constructs, reliable classifiers can be used in an early phase as a filter on the complete dataset to create a small subdataset that can be coded by hand.

The current study introduced two possible forms of biases. First, our sample consisted of posts from one forum on a highly trusted Dutch cancer website. Users on this forum might differ from the general cancer population in that they must have the skills to go online and register before using this forum. Furthermore, the fact that these patients opened a forum post could be an indication that they experienced a problem during a previous communication (eg, a shortage of information or conflicting information during the consultation with their medical expert). Therefore, the results might not be representative of all cancer patients, and the needs and motives found could be an overestimation of the unmet needs in this population. However, a complete export of all of the content of a platform with the informed consent of all users is still difficult to obtain, thus illustrating the uniqueness of our study. While the reported unmet needs might be an overestimation, these unmet needs still exist and will likely continue to exist. Therefore, scholars, medical experts, and (cancer) patient associations should work together to make convergence as easy as possible and try to incorporate alternative sources of information into the medical trajectory. For example, a leaflet or a website hosted by the hospital can provide patients with reliable sources but also well-known forums in which patients can exchange experiences and find support.

The second possible bias could have been created during the SML process. The SML algorithm that was used to create a sample of the posts used for the analysis might have caused a bias in the reference category. We manually created the reference category in which no signs of convergence were present. However, it is possible that the original algorithm marked these posts as false positives based on some shared content characteristics. This process might have led to differences between these false positives and the posts without signs of convergence in the corpus (ie, dataset) that were left out of the analysis. As a result, the reference sample might not completely be representative of the posts without signs of convergence. However, most of the main results are from a comparison between mass and interpersonal communication. These two samples were created by a combination of SML and manual checking; therefore, the above-described bias does not play a role. To overcome this possible bias, future research could either randomly create a sample as the reference category or possibly compare the reference category that was created through machine learning to a random sample before running the analysis.

### Conclusions

To conclude, convergence is an important concept that represents the natural flow of patients’ information-seeking behavior between and within interpersonal and mass communication. Understanding how patients use different communication channels is essential to improving health care by providing guidance to patients who are trying to fulfill their needs. A better understanding of the conditions (ie, whether the information is discussed and in which way) under which the convergence of interpersonal and mass media results in positive patient outcomes might be the key to enhancing information provision to patients and in turn increasing patients’ wellbeing. In doing so, providers should take a proactive role in discussing online information-seeking with patients and referring patients to the right sources that best meet their needs.

## Appendix

Multimedia Appendix 1 Details of the supervised machine learning (SML) process.

Multimedia Appendix 2 Confusion matrix classifiers per category.

## Abbreviations

OR: odds ratio
SML: supervised machine learning
