# Supplementary material for: Patients’ Convergence of Mass and Interpersonal Communication on an Online Forum: Hybrid Methods Analysis
Source: J Med Internet Res. 2020 Oct 19;22(10):e18303. doi: 10.2196/18303 (PMC7605979; doi:10.2196/18303)
Supplement: Multimedia Appendix 1 [file jmir_v22i10e18303_app1.docx]

**Multimedia Appendix 1**

We used SML to train classifiers that could automatically detect the categories established in the manual content analysis. This was done in multiple stages, as we followed an iterative process that checked the performance of the classifiers at reach step. The quality of the classifiers was assessed based on precision, recall, and F1 scores for their predictions of cases in which the category was present (i.e., for cases in which the reference to mass or interpersonal media was 1. Precision gives the proportion of the automatically assigned labels that correspond with the human-labeled data. Recall gives the proportion of the true labels that are found automatically. As a result, there is often a tradeoff between the scores of precision and recall; for example, when you assume a higher recall, the chance that some of the recalled data are false positive grows, and as a result, the precision score suffers. F1 scores are the harmonic mean of the recall and precision.

The first sample of SML consisted of 306 manually-labeled posts. The dataset was split into a training set and a test set using an 80-20 split *(N_train_=244; N_test_= 62).* Using *Scikit-Learn* [44], the data were preprocessed and various classifiers were trained using different algorithms, such as support vector classification (SVC), stochastic gradient descent (SGD), multinomial naïve Bayes (MNB), gradient boosting, and passive aggressive classifier, to evaluate which algorithm had the best performance for each classifier. Given the relatively small dataset, we also assessed whether using a k-fold cross-validation technique (a method in which the data are partitioned in “k” datasets and analyses are performed on the subsets of the data) would improve the performance of the classifiers, however it did not yield positive results and was discarded.

We adopted a *gridsearch* strategy to search for the best estimation technique and parameter settings. Parameters to be tested included, ngram range, the use of tfidf, class weight (i.e., balanced, none), loss (e.g., hinge, log, modified huber, perceptron, deviance, exponential) and alpha (e.g. 1, 0.1, 0.01, 0,001).

The initial results of the SML showed a good fit for the broad classifiers of the signs of convergence and whether an online or offline mass medium was referenced (*F1*_avr_ = 0.89 - 0.93). However, this estimated was skewed due to the large number of cases in which the manually coded data was labeled as *not present* (0). Furthermore, few occurrences for the interpersonal media specification classifiers were found; as a result, no suitable SML algorithm could be developed.

To further improve the quality of all the classifiers, an additional random sample of 379 posts was manually coded and divided, again, in a training and test set*(N_train_=548; N_test_= 137)*. SML was applied again on the larger sample (*N* = 685). The results of the final SML are displayed in Table 5. The classifiers from the category *convergence:* *mass communication* and *interpersonal communication*, were deemed reliable enough use. Since the classifiers from other categories showed mixed results, these categories were discarded from further steps and were manually-coded in a later phase instead**.** Using the classifiers from the category convergence, a subsample of posts likely to contain signs of convergence was created by predicting the complete sample of opening posts.

Predicting the complete dataset of opening posts (*N* = 1,708) showed that 45,14% of the posts were automatically-labeled as having signs of convergence (*N* = 771). To ensure validity, the automatically-labeled convergence classifiers were evaluated by hand. A total of 245 posts (31,78%) did not contain signs of convergence after all and was coded as such. The subsample (*N* = 771) were coded using the codebook by the first author (see Table 1).
