# Supplementary material for: Patients’ Convergence of Mass and Interpersonal Communication on an Online Forum: Hybrid Methods Analysis
Source: J Med Internet Res. 2020 Oct 19;22(10):e18303. doi: 10.2196/18303 (PMC7605979; doi:10.2196/18303)
Supplement: Multimedia Appendix 2 [file jmir_v22i10e18303_app2.docx]

**Multimedia Appendix 2**. Confusion matrix classifiers per category.

| Concept | | Best performing classifier | Recall (1) | Precision (1) | F1 (1) | Decision |
| --- | --- | --- | --- | --- | --- | --- |
| Convergence | |  |  |  |  |  |
|  | Mass media | SGD^a^ | 0.86 | 0.92 | 0.89 | Used in the full sample |
|  | Interpersonal media | SGD | 0.76 | 0.96 | 0.85 | Used in the full sample |
| Specification of convergence | |  |  |  |  |  |
|  | Medical expert | SGD | 0.67 | 0.21 | 0.32 | Not used in the full sample |
|  | Others | SGD | 0.25 | 0.17 | 0.20 | Not used in the full sample |
|  | Mass media, online | SGD | 0.76 | 0.38 | 0.51 | Not used in the full sample |
|  | Mass media, offline | SGD | 0.00 | 0.00 | 0.00 | Not used in the full sample |
| Motive for posting | |  |  |  |  |  |
|  | Conflict | SGD | 0.71 | 0.16 | 0.26 | Not used in the full sample |
|  | Shortage of information | SGD | 0.00 | 0.00 | 0.00 | Not used in the full sample |
|  | New question | SGD | 0.46 | 0.11 | 0.18 | Not used in the full sample |
|  | Sharing information | SGD | 0.86 | 0.50 | 0.63 | Not used in the full sample |
| Information on poster | |  |  |  |  |  |
|  | Disease or treatment information | SGD | 0.88 | 0.82 | 0.85 | Not used in the full sample |
|  | Time indication disease or treatment | Passive | 0.94 | 0.73 | 0.82 | Not used in the full sample |
|  | Type of cancer | SGD | 0.93 | 0.65 | 0.77 | Not used in the full sample |
|  | Cancer in the surrounding community | SGD | 0.92 | 0.39 | 0.55 | Not used in the full sample |
| Needs | |  |  |  |  |  |
|  | Community building | SGD | 0.71 | 0.71 | 0.71 | Not used in the full sample |
|  | Sharing experience | SGD | 0.56 | 0.27 | 0.36 | Not used in the full sample |
|  | Asking experience | SGD | 0.89 | 0.91 | 0.90 | Not used in the full sample |
|  | Asking information | SGD | 0.50 | 0.07 | 0.12 | Not used in the full sample |

^a^SGD: stochastic gradient descent.
